# Risk Factors and Clinical Characteristics of Neonatal Acute Respiratory Distress Syndrome Caused by Early Onset Sepsis

**DOI:** 10.3389/fped.2022.847827

**Published:** 2022-03-28

**Authors:** Ting You, Yan-Rong Zhou, Xiao-Chen Liu, Lu-Quan Li

**Affiliations:** ^1^Department of Neonatology, National Clinical Research Center for Child Health and Disorders, Ministry of Education Key Laboratory of Child Development and Disorders, Chongqing Key Laboratory of Pediatrics, Children’s Hospital of Chongqing Medical University, Chongqing, China; ^2^Jiulongpo People’s Hospital of Chongqing, Chongqing, China

**Keywords:** early onset sepsis, acute respiratory distress syndrome, clinical characteristics, risk factors, neonate

## Abstract

**Purpose:**

To identify risk factors associated with the development of acute respiratory distress syndrome (ARDS) in infants with early onset sepsis (EOS) and to describe the clinical features.

**Methods:**

A retrospective study was conducted at the Children’s Hospital of Chongqing Medical University between January 2000 and October 2020. The infants were divided into ARDS and non-ARDS groups. Clinical characteristics and risk factors were compared between the two groups.

**Results:**

Two hundred fifty infants (58 with ARDS) were included. Smaller gestational age, lower birth weight (LBW), lower serum albumin level, a higher rate of preterm birth, premature rupture of membranes, antenatal steroid exposure, and lower Apgar score were associated with an increased development of ARDS by univariate analysis (*P* < 0.05). LBW (β = −0.001, *P* = 0.000, *OR*: 0.999, 95% *CI*: 0.998–0.999) and low serum albumin levels (β = −0.063, *P* = 0.022, *OR*: 0.939, 95% *CI*: 0.889–0.991) were identified as independent risk factors for the development of ARDS by logistic regression analysis. A higher frequency of complications, including persistent pulmonary hypertension, intraventricular hemorrhage, pulmonary hemorrhage, septic shock, and bronchopulmonary dysplasia, was found in the ARDS group (*P* < 0.05). The rate of mortality was higher for those in the ARDS group than for those in the non-ARDS group (46.6% vs. 15.6%, χ^2^ = 24.205, *P* = 0.000).

**Conclusion:**

Acute respiratory distress syndrome (ARDS) in EOS could lead to a higher frequency of complications and mortality. The risk factors for the development of ARDS were LBW and low serum albumin levels.

## Introduction

Although advances in medical technology have improved over the past several decades, early onset sepsis (EOS), defined as sepsis occurring within 72 h after birth, still remains one of the most common causes of neonatal morbidity and mortality (1). The survivors of EOS have a high incidence of various complications and poor outcomes in very low birth weight infants (2). EOS may present just as a gradual onset of signs (such as lethargy, apnea, cyanosis, hypo- or hyperthermia, and poor feeding) or as rapid progression featuring acute respiratory distress syndrome (ARDS) soon after birth (3, 4). The characteristics of sepsis complicated with ARDS in adults and children have been well studied (5–7) and yield greater complications, such as neurological dysfunction, septic shock, renal injury, and cardiac damage. In addition, ARDS is correlated with high mortality in infants with sepsis (7–9). In neonates, previous studies have shown that early onset *group B Streptococcus* (GBS) infection can manifest as ARDS (3, 10) and may be associated with the highest case-fatality rate (11, 12).

In addition to GBS, organisms such as *Klebsiella pneumoniae*, *Escherichia coli*, *Staphylococcus aureus*, and *Listeria monocytogenes* are involved in EOS (13). Thus, a more comprehensive understanding of the risk factors and clinical features of EOS complicated with ARDS is still needed to develop better treatment strategies. The aim of this study was to identify the risk factors associated with the development of ARDS in EOS infants and to describe their clinical features.

## Materials and Methods

### Study Population

This retrospective study was conducted at the Children’s Hospital of Chongqing Medical University (CHCMU) between January 2000 and October 2020. The infants who were diagnosed with EOS were included in this study. EOS was defined as a sepsis occurring within 72 h of birth (14). The definition of sepsis was proven by positive blood or cerebrospinal fluid culture with clinical manifestations such as lethargy, hypothermia (<36.5°C), poor feeding, apnea [a pause of breathing for more than 15–20 s or accompanied by oxygen desaturation (peripheral capillary oxygen saturation ≤80%) and bradycardia], tachypnea (an increase in the sinus rate of above 160–180 beats/min), grunting, nasal flaring, cyanosis, desaturation (peripheral capillary oxygen saturation <85%), bradycardia (heart rate <100 bpm for more than 5 s), and poor perfusion, which were in accordance with the diagnostic criteria of EOS (15). Two successive positive blood cultures with the same antibiotic susceptibility pattern were required for the diagnosis of *coagulase-negative Staphylococcus* infection. Neonatal ARDS was defined using the Montreux definition published in 2017 (16). The diagnostic criteria of intraventricular hemorrhage (IVH) were graded according to the Papile criteria (17). This study received an ethics approval from the Institutional Review Board of CHCMU (No: 2016-16), and the use of the database housing the evaluated data was permitted by the ethics committees of CHCMU.

### Inclusion and Exclusion Criteria

The inclusion criteria included the infants who had positive blood or cerebrospinal fluid cultures with clinical manifestations of sepsis, in accordance with the diagnostic criteria of neonatal sepsis (15), and infants who were admitted within 24 h after birth. The exclusion criteria included the infants with incomplete records or a lack of infectious clinical manifestations, although they were combined with a positive blood culture.

### Data Collection

Medical record data were extracted from the medical record management system and included the gender, birth weight, gestational age (GA), Apgar score, mode of delivery and pregnancy-induced hypertension, diabetes, prenatal corticosteroids, intrahepatic cholestasis of pregnancy, a premature rupture of membranes (PROM) ≥18 h, and chorioamnionitis. The information on laboratory tests (within 24 h after birth), clinical complications, therapeutic strategies, and outcomes were also reviewed.

### Statistical Analysis

All analyses were performed by the SPSS statistical software (version 17; SPSS, Chicago, IL, United States). Continuous data were reported as means and standard deviations and were analyzed by means of *Student’s t-test*. Non-normally distributed measurement data were presented as the median (M) and interquartile range (IQR) and were analyzed by means of the *Wilcoxon rank-sum test*. Categorical data were reported as counts and percentages, and chi-square tests were used to compare characteristics. Logistic regression analyses were performed to determine the risk factors for the development of ARDS. *P* < 0.05 was considered statistically significant.

## Results

### Baseline Information

**FIGURE 1:**
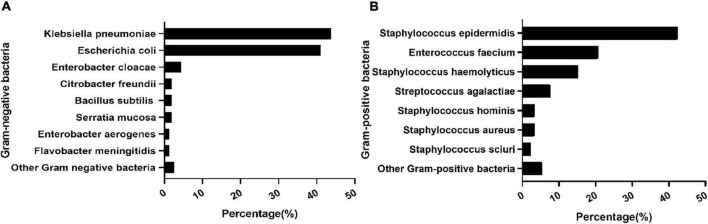
Distribution of pathogens. Composition of major Gram-positive bacteria **(A)**. Composition of major Gram-negative bacteria **(B)**.

During the study period, 273 infants had positive cultures in the Neonatal Diagnosis and Treatment Center of CHCMU. Among them, twenty-three were excluded from further study due to incomplete records (*n* = 3) and the contamination of blood samples (*n* = 20). Therefore, 250 cases were included in the final analysis. A total of 58 (23.20%) infants had cases complicated with ARDS, 96 (38.4%) infants were preterm, and 80 (32.0%) infants had low birth weight (LBW). PROM and chorioamnionitis were found in 59 (23.6%) and 46 (18.4%) infants, respectively, and antenatal steroid exposure was found in 33 (13.2%) infants. *K. pneumoniae* (27.6%), *E. coli* (26.0%), and *S. epidermidis* (15.6%) remain the principal organisms responsible for EOS. Among the Gram-negative bacteria (GNB), *K. pneumoniae* (43.7%) and *E. coli* (41.1%) were the main pathogens. Meanwhile, among Gram-positive bacteria (GPB), *S. epidermidis* (54.2%), *Enterococcus faecium* (20.7%), and *S. haemolyticus* (15.2%) were major pathogens (Figure 1).

### Identification of Risk Factors for the Development of Acute Respiratory Distress Syndrome in Early Onset Sepsis Infants by Univariate Analysis

In order to determine which factors are related to the occurrence of ARDS, we compared the baseline data and laboratory results of the two groups. Compared with infants without ARDS, infants with ARDS had LBW, a higher rate of preterm birth, PROM, and antenatal steroid exposure and a lower Apgar score (*P* < 0.05). Other parameters, such as gender and the mode of delivery, were not significantly different between the two groups (Table 1). Meanwhile, low serum albumin levels were found in infants with ARDS (*P* < 0.05), and other hematologic parameters, such as white blood cell counts, immature/total neutrophil ratio, C-reactive protein, platelet counts, and hemoglobin concentrations showed no significant difference between the ARDS and non-ARDS groups (Table 2).

**TABLE 1:** Demographic characteristics of infants enrolled in the present study.

Variable	ARDS (*n* = 55)	Non-ARDS (*n* = 195)	Statistic	*P*
Gestational age, M (IQR), w	33.86 (31.29–38.00)	38.57 (36.43–39.86)	5.390	0.000
Birth weight, M (IQR), g	2,000 (1,510–2,800)	2,995 (2,519–3,400)	5.268	0.000
Apgar 1 min, M (IQR)	8 (7–9)	9 (8–10)	2.744	0.006
Apgar 5 min, M (IQR)	9 (8–10)	10 (9–10)	4.014	0.000
Male,% (*n*)	49.1 (27)	60.5 (118)	2.298	0.130
Age of admission, M (IQR), h	0.85 (0.08–4.67)	4.97 (2.11–8.53)	3.975	0.000
Low birth weight,% (*n*)	61.8 (34)	24.1 (47)	27.862	0.000
Prematurity,% (*n*)	70.9 (39)	29.2 (57)	31.504	0.000
Meconium-stained amniotic fluid,% (*n*)	16.4 (9)	19.0 (37)	0.195	0.659
PROM,% (*n*)	43.6 (24)	17.9 (35)	15.700	0.000
Maternal hypertension,% (*n*)	5.5 (3)	4.1 (8)	0.004	0.953
GDM,% (*n*)	9.1 (5)	8.2 (16)	0.000	1.000
Antenatal steroid use,% (*n*)	30.9 (17)	8.2 (16)	19.300	0.000
Cesarean section, *n* (%)	50.9 (28)	46.7 (91)	0.310	0.578
Twins, *n* (%)	34.5 (19)	17.9 (35)	0.978	0.008
ICP, *n* (%)	3.6 (2)	1.5 (3)	0.190	0.663
Total volume within 24 h after birth, (± SD), ml/kg	84.41 ± 15.18	81.39 ± 17.23	0.914	0.473

*PROM: premature rupture of membranes >18 h; GDM: gestational diabetes mellitus; ICP: intrahepatic cholestasis of pregnancy; M: median; IQR: interquartile range.*

**TABLE 2:** Comparison of laboratory results between the two groups of infants.

Variable	ARDS (*n* = 55)	Non-ARDS (*n* = 195)	Statistic	*P*
Albumin, M (IQR), g/L	27.35 (24.70–32.63)	32.60 (28.30–36.60)	3.998	0.000
WBC < 5 or > 30 (× 10^9^/L),% (n)	30.8 (16)	21.2 (41)	2.082	0.149
Hemoglobin (± SD), g/L	144.1 ± 30.51	153.23 ± 36.08	1.653	0.100
I/T > 0.16,% (*n*)	13.7 (7)	11.9 (20)	0.011	0.918
Platelet count < 100 × 10^9^/L,% (*n*)	21.2 (11)	20.7 (40)	0.005	0.946
CRP > 10 mg/L,% (*n*)	42.3 (22)	43.6 (79)	0.029	0.864
Gram-negative bacteria,% (*n*)	69.1 (38)	62.1 (121)	0.918	0.388

*WBC: white blood cell count; I/T: immature/total neutrophil; CRP: C-reactive protein; M: median; IQR: interquartile range.*

### Identification of Independent Risk Factors of Acute Respiratory Distress Syndrome in Early Onset Sepsis Infants by Multivariate Analysis

**FIGURE 2:**
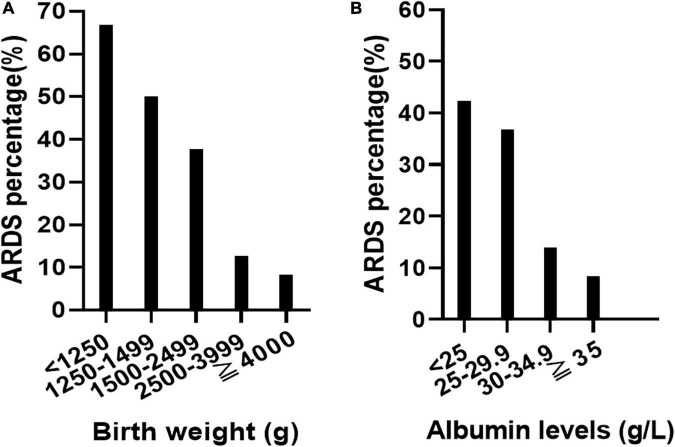
Relationship between birth weight, serum albumin level, and the incidence of acute respiratory distress syndrome (ARDS). With the decrease of birth weight, the incidence of ARDS increased gradually **(A)**. With the decrease of plasma albumin level, the incidence of ARDS increased gradually **(B)**.

Low birth weight (β = −0.001, *P* = 0.000, *OR*: 0.999, 95% CI: 0.998–0.999) and low serum albumin levels (β = −0.063, *P* = 0.022, *OR*: 0.939, 95% CI: 0.889–0.991) were independently associated with an increased morbidity of ARDS in EOS infants by logistic regression analysis. The development of ARDS at different birth weights and serum albumin levels is shown in Figure 2.

### Clinical Complications in Infants With Acute Respiratory Distress Syndrome

Overall, significantly higher rates of complications, including persistent pulmonary hypertension (PPHN), IVH, pulmonary hemorrhage, hypoglycemia, septic shock, renal insufficiency, bronchopulmonary dysplasia (BPD), and coagulation disorders, were found in the ARDS group (*P* < 0.05) (Table 3).

**TABLE 3:** Comparison of neonatal complications between the two groups of infants.

Variable	ARDS (*n* = 55)	Non-ARDS (*n* = 195)	Statistic	*P*
IVH,% (*n*)	40.0 (22)	15.4 (30)	15.779	0.000
PPNH,% (*n*)	30.9 (17)	5.6 (11)	27.540	0.000
BPD,% (*n*)	7.3 (4)	0.5 (1)	6.850	0.009
Septic shock,% (*n*)	16.4 (9)	3.1 (61)	11.176	0.001
Pulmonary hemorrhage,% (*n*)	21.8 (12)	1.5 (3)	27.790	0.000
NEC,% (*n*)	7.3 (4)	11.8 (23)	0.921	0.340
Hypoglycemia,% (*n*)	21.8 (12)	7.7 (15)	8.886	0.003
Renal insufficiency,% (*n*)	40.0 (22)	12.3 (24)	21.911	0.000
Coagulation dysfunction,% (*n*)	58.1 (32)	29.7 (58)	15.058	0.000
Heart failure,% (*n*)	3.6 (2)	0.0 (0)	–	0.048

*IVH: intraventricular hemorrhage; PPHN: persistent pulmonary hypertension; BPD: bronchopulmonary dysplasia; NEC: necrotizing enterocolitis.*

### Antimicrobial Susceptibility to Different Types of Pathogens of Early Onset Sepsis-Related Acute Respiratory Distress Syndrome

No statistically significant difference was found in most antibiotic resistance rates between the two groups. GNB showed a low degree of sensitivity to common antibiotics, such as penicillin (21.8%), beta-lactams (45.2%–73.5%) and cephalosporins (31.1–59.7%), while a relatively high sensitivity to quinolones (86.1–87.8%) and aminoglycosides (76.8–96.5%) was observed among GNB. Furthermore, in this study, in a cohort of newborns with GNB, the incidence of ESBL was 53.3% (Table 4).

**TABLE 4:** Rates of Gram-negative bacteria susceptibility to different antibiotics [% (*n*)].

Variable	Total (*N* = 159)	ARDS (*N* = 38)	Non-ARDS (*N* = 121)	Statistics	*P*
**Penicillin**					
Piperacillin	21.8 (26)	15.4 (4)	23.7 (22)	0.814	0.367
**Beta-lactam**					
Ampicillin + Sulbactam	45.2 (66)	48.6 (17)	44.1 (49)	0.211	0.646
Piperacillin + Tazobactam	73.5 (108)	72.2 (26)	73.9 (82)	0.038	0.845
**Cephalosporin**					
Cefazolin	31.1 (47)	33.3 (12)	30.4 (35)	0.107	0.743
Ceftazidime	59.7 (92)	55.6 (20)	61.0 (72)	0.342	0.559
Cefotaxime	39.9 (61)	30.6 (11)	42.7 (50)	1.703	0.192
**Carbapenem**					
Imipenem	91.9 (143)	91.7 (33)	90.9 (110)	0.00	1.000
**Quinolone**					
Levofloxacin	87.8 (130)	75.0 (27)	92.0 (103)	5.837	0.016
Ciprofloxacin	86.1 (130)	77.8 (28)	88.7 (102)	2.730	0.099
**Aminoglycoside**					
Amikacin	96.5 (138)	94.3 (33)	97.2 (105)	0.676	0.770
Gentamicin	76.8 (116)	63.9 (23)	80.9 (93)	4.440	0.035
**Others**					
ESBL (+),% (*n*)	53.3 (72)	66.7 (22)	49.0 (50)	3.120	0.077

*Partial strains were not tested for sensitivity to this drug; the denominator inside parentheses comprises the actual bacterial strains tested for sensitivity to this drug. ESBL: extended-spectrum β-lactamases.*

The total antibiotic resistance rates to the following drugs were high among GPB strains: ampicillin (63.8%), erythromycin (70.1%), azithromycin (68.4%), and amoxicillin-clavulanic acid (74.4%). However, all GPBs were sensitive to vancomycin, teicoplanin, linezolid, and tigecycline (Table 5).

**TABLE 5:** Rates of gram-positive bacteria susceptibility to different antibiotics [% (*n*)].

Variable	Total (*N* = 91)	ARDS (*N* = 14)	Non-ARDS (*N* = 79)	Statistics	*P*
**Penicillin**					
Ampicillin	36.2 (17)	62.5 (5)	30.8 (39)	1.684	0.194
**Glycopeptide**					
Teicoplanin	100.0 (74)	100.0 (12)	100.0 (62)	–	–
Vancomycin	100.0 (88)	100.0 (14)	100.0 (71)	–	–
**Macrolides**					
Azithromycin	31.6 (12)	33.3 (2)	31.3 (10)	0.000	1.000
Erythromycin	29.9 (26)	33.3 (5)	29.2 (21)	0.000	0.991
**Quinolone**					
Levofloxacin	61.8 (40)	64.3 (9)	68.9 (31)	0.000	1.000
Ciprofloxacin	61.8 (47)	61.5 (8)	61.9 (39)	0.000	1.000
**Aminoglycoside**					
Amikacin	58.8 (20)	62.5 (5)	57.7 (15)	0.000	1.000
Gentamicin	70.5 (55)	62.2 (9)	70.8 (46)	0.000	1.000
**Oxazolidinones**					
Linezolid	100.0 (84)	100.0 (14)	100.0 (76)	–	–
**Others**					
Tigecycline	100.0 (47)	100.0 (9)	100.0 (38)	–	–
Amoxicillin-clavulanic acid	25.6 (20)	28.6 (4)	25.0 (16)	0.000	1.000
Rifampicin	86.6 (58)	88.9 (8)	86.2 (50)	0.000	1.000

*Partial strains were not tested for sensitivity to this drug; the denominator inside parentheses comprises the actual bacterial strains tested for sensitivity to this drug.*

All infants were treated with antibiotics according to drug sensitivity. The rate of mortality was higher for those in the ARDS group than for those in the non-ARDS group (46.6 vs. 15.6%, χ^2^ = 24.205, *P* = 0.000). In infants with ARDS, no significant difference in the use of a pulmonary surfactant (PS) between the survivor group and the non-survivor group was found (53.6 vs. 46.4%, χ^2^ = 0.000, *P* = 0.986).

## Discussion

In recent years, with the rapid development of maternal fetal medicine and neonatal intensive care medicine, the ARDS of neonates has received increasing attention (18). ARDS has long been recognized as a devastating complication of sepsis (19). We found that ARDS developed rapidly in EOS infants and was associated with more complications and higher mortality. Therefore, identifying early independent risk factors for the development of ARDS in EOS infants might be helpful for optical therapeutic strategies.

### Risk Factors for the Development of Acute Respiratory Distress Syndrome in Early Onset Sepsis Infants

Our findings suggested that LBW and low serum albumin levels are independent risk factors for the development of ARDS in EOS infants. The incidence of ARDS increased gradually with decreasing birth weight in the present study. ARDS has long been recognized as a devastating complication of sepsis (19); it develops because sepsis can initiate a systemic inflammatory response that releases proinflammatory cytokines such as interleukin 6 (IL-6), IL-1β, IL-8, and tumor necrosis factor, and high levels of these cytokines can damage the alveolar-capillary barrier and alveolar type II cells (20, 21). Then, plasma, large proteins, and cellular blood components leak from the pulmonary capillary into the lung tissues and cause acute pulmonary edema, which accelerates the inactivation of PS (22–24). Meanwhile, oxidation and hydrolysis activated by inflammatory reactions can increase the degradation of PS, eventually leading to hyaline membrane formation and alveolar collapse (25). The lower the birth weight, the less mature the lung type II epithelial cells, and the less PS secreted, which will seriously hinder the expansion of the lung, leading to severe respiratory distress.

Low serum albumin levels were also independently associated with the development of ARDS in EOS infants in the current study. The association between low serum albumin levels and the development of ARDS in EOS infants may be due to the following reasons. First, low serum albumin levels are a marker of malnutrition (18). Malnutrition leads to respiratory muscle dysfunction, which decreases oxygenation (26, 27). Second, serum albumin plays an essential role in maintaining the regulation of intravascular volume and fluid balance (28, 29). Low serum albumin levels cause a lower lung oncotic pressure, which contributes to the leakage of fluid into the pulmonary interstitial tissues and leads to a decrease in lung compliance, inefficient gas exchange, and the inactivation of PS (30, 31). Furthermore, it has been postulated that albumin has the antioxidant capability of scavenging free radicals and preventing apoptosis in the respiratory tract (31). Hence, low serum albumin levels reduce the ability to combat oxidative stress and further increase the risk of lung oxidative injury, which increases the degradation of PS and eventually leads to the development of ARDS (18, 30, 32, 33).

Other potential possible risk factors for ARDS in EOS infants include preterm birth, PROM, and a low Apgar score. PROM increases the risk of intrauterine infection, which may lead to systemic fetal inflammatory response syndrome (FIRS) (34). FIRS has been implicated as a cause of long-term fetal lung and eventually drives the development of ARDS (35). A low Apgar score might also be responsible for the development of ARDS in EOS infants. Animal experiments have shown that hypoxia can reduce the content of phospholipids and PS protein and decrease the blood vessel density of alveolar interstitial tissues in fetal rats (36, 37), which can cause vasospasm and lead to an increase in pulmonary capillary permeability and the formation of hyaline membranes. Asphyxia at birth can directly damage alveolar type II epithelial cells, reduce PS production, and inhibit the activity of PS (38).

### High Complications and Mortality of Acute Respiratory Distress Syndrome in Early Onset Sepsis Infants

Early onset sepsis could increase the risk of respiratory failure, IVH, pulmonary hemorrhage, and severe retinopathy of prematurity (1, 14, 39). In this study, we further found that infants with ARDS had more multiple organ complications and more than threefold higher mortality than infants who did not develop ARDS. Similar high mortality was observed in a previous study in adults or children with sepsis-related ARDS (7, 8). Higher occurrence rates of IVH, PPHN, BPD, coagulation disorders, hypoglycemia, renal insufficiency, septic shock and pulmonary hemorrhage were found in infants with ARDS. The possible explanation is that ARDS leads to more severe lung compliance reduction, low gas exchange efficiency, and increased physiological dead space, which lead to multiple organ hypoxia and dysfunction.

### Low Sensitivity to the Most Commonly Used Antibiotics in Early Onset Sepsis Infants With Acute Respiratory Distress Syndrome

In the current study, *K. pneumoniae*, *E. coli*, and *S. epidermidis* remained the principal organisms responsible for EOS. This result is similar to the previously reported pathogen composition of EOS in China (40), while it seemed to differ from those in developed countries whose main pathogens of EOS were *E. coli* and GBS (1, 41, 42). This difference might be partly attributed to different population characteristics and intrapartum/postnatal healthcare strategies (43).

Our study showed that the majority of GNBs had high resistance to commonly used antibiotics, such as semisynthetic penicillin and cephalosporins, and exhibited the highest sensitivity rates to imipenem and amikacin. GPB showed a high resistance to penicillin and erythromycin. Teicoplanin and vancomycin were the most efficacious drugs in our study, which was similar to a previous study (7). However, a study from the Eunice Kennedy Shriver National Institute of Child Health showed that ampicillin and gentamicin remained effective antibiotics in most EOS cases in western countries (42). This significant difference might be attributed to (1) the overuse and misuse of broad-spectrum antibiotics, particularly cephalosporins, in neonates in China (45) and (2) the routine feeding of antibiotics to healthy farm animals, which occurs without a prescription and promotes the development of antibiotic-resistant bacteria that can be transferred to humans (46).

There are some limitations in this study, including the errors and bias inherent to the nature of a retrospective study. Additionally, the number of subjects was relatively small in this single-center study, and multicenter studies are therefore recommended. In this study, the GA of some infants was less than 34 weeks, which was also a risk factor for neonatal respiratory distress syndrome (NRDS). Therefore, it is difficult to completely rule out the possibility of NRDS + EOS in some infants. However, the application of PS did not improve the prognosis of these infants with respiratory distress, which was obviously inconsistent with the consensus that PS has a significant effect on NRDS (47).

## Conclusion

In summary, LBW and low serum albumin levels are associated with an increased development of ARDS in EOS infants. EOS with ARDS is associated with a higher overall disease severity, more severe complications, and higher case-fatality rate.

## Data Availability Statement

The raw data supporting the conclusions of this article will be made available by the authors, without undue reservation.

## Ethics Statement

The studies involving human participants were reviewed and approved by the Institutional Review Board of Children’s hospital of Chongqing Medical University. Written informed consent from the participants’ legal guardian/next of kin was not required to participate in this study in accordance with the national legislation and the institutional requirements.

## Author Contributions

TY contributed to the acquisition, analysis and interpretation of the data and the drafting, and final approval of the manuscript. Y-RZ contributed to the acquisition, analysis and interpretation of the data. X-CL contributed to the acquisition of the data. L-QL supervised the project and contributed to the conception and design of the study, analysis and interpretation of the data, and critical revision and final approval of the manuscript. All authors made substantial contributions to the study and manuscript and met the criteria for authorship defined in the author instructions and reviewed the manuscript, provided feedback and approved the manuscript in its final form.

## Conflict of Interest

The authors declare that the research was conducted in the absence of any commercial or financial relationships that could be construed as a potential conflict of interest.

## Publisher’s Note

All claims expressed in this article are solely those of the authors and do not necessarily represent those of their affiliated organizations, or those of the publisher, the editors and the reviewers. Any product that may be evaluated in this article, or claim that may be made by its manufacturer, is not guaranteed or endorsed by the publisher.
